# Association of peripheral BDNF level with cognition, attention and behavior in preschool children

**DOI:** 10.1186/s13034-016-0097-4

**Published:** 2016-05-19

**Authors:** Chan-Woo Yeom, Young-Ja Park, Sam-Wook Choi, Soo-Young Bhang

**Affiliations:** Department of Psychiatry, Bugok National Hospital, Changnyeong-gun, Gyeongsangnam-do South Korea; Department of Internal Medicine, Ulsan University Hospital, Ulsan, South Korea; Korea Institute on Behavioral Addictions, Easy Brain Clinic, Seoul, South Korea; Health Care and Information Research Institute, Namseoul University, Cheonan, South Korea; Department of Psychiatry, Eulji University School of Medicine, Eulji General Hospital, 68 Hangeulbiseok-Ro, Nowon-Gu, Seoul, 139-711 South Korea

**Keywords:** BDNF, Brain-derived neurotrophic factor, Cognition, ADHD, Neurodevelopment

## Abstract

**Background:**

Brain-derived neurotrophic factor (BDNF) has been reported to affect development, cognition, attention and behavior. However, few studies have investigated preschool children with regard to these areas. We evaluated the relationship between cognition, attention and peripheral blood concentration of BDNF in preschool children.

**Methods:**

Twenty-eight children (mean age: 6.16 ± 0.60 years) were recruited. For all subjects, serum and plasma BDNF levels were assessed; intelligence was assessed using the Korean standardisation of the Wechsler Intelligence Scale for Children (KEDI-WISC); attention was assessed using the computerised continuous performance test (CCPT), the children’s color trails test (CCTT), the Stroop color-word test for preschool children, and the attention-deficit/hyperactivity disorder rating scale (K-ARS); and finally emotional and behavioral problems were assessed using the child behavior checklist (K-CBCL). We confirmed the previously reported correlations between the various psychometric properties assessed and serum and plasma levels of BDNF in our sample.

**Results:**

Serum BDNF levels were negatively correlated with both KEDI-WISC full scale IQ (FSIQ, r = −0.39, p = 0.04) and verbal IQ (VIQ, r = −0.05, p = 0.01), but not with the performance IQ (PIQ, r = −0.12, p = 0.56). There were no significant relationships between plasma BDNF level and VIQ, PIQ or FSIQ. No correlations were found between either serum or plasma level of BDNF and any of the attentional measures (CCPT, ARS, CCTT or Stroop color word test). The CBCL total behavioral problem and attention problem sections were positively correlated with plasma BDNF level (r = 0.41, p = 0.03), (r = 0.44, p = 0.02), however, no relationship was found between the serum BDNF and any of the composite CBCL measures.

**Conclusions:**

Our results suggest that high peripheral BDNF may be negatively correlated with intelligence, behavioral problems and clinical symptoms of neuro-developmental disorders such as intellectual disability in preschool children. A high peripheral BDNF concentration may, if these findings are further replicated, prove to be a useful biomarker for such issues in preschool children.

## Background

Brain-derived neurotrophic factor (BDNF) is a member of the neurotrophin family, which is expressed in human and other mammalian brains [1]. BDNF is associated with synaptic plasticity, synaptic connectivity formation and neuronal survival [2–4]. It also serves an important role during brain development [3] through the regulation of neural circuit development by selective embryonic neural stem cell survival and differentiation, axonal growth and guidance, synapse formation and maturation, and the refinement of developing circuits [4].

BDNF plays an important role in learning and memory [5–7]. Several reports relate BDNF levels to task performance in cognitive assessment of the rat [8–10]. Administration of BDNF enhances rat performance in the Morris water maze (MWM) [8], while injection of BDNF antibodies into their lateral ventricles is correlated with poorer MWM performance [9]. However, Cunha et al. [10] have suggested that chronic BDNF over-expression in young adult transgenic mice (9–14 week) can induce learning deficits and short-term memory impairment on both spatial and instrumental learning tasks.

A number of reports suggest a relationship between BDNF and the functioning of certain brain areas involved in attention and cognition [11–15]. The highest levels of CNS BDNF are found in the hippocampus, frontal cortex, and amygdale [11, 12]. Both endogenous BDNF and intra-hippocampal BDNF infusion induce hippocampal long-term potentiation, which is critical to the physiology of long-term memory formation [13, 14]. BDNF plays an important role in the working memory of the prefrontal cortex [15].

Currently, most research on the interrelationships between mood, psychosis, cognition, attention and peripheral BDNF concentration has been conducted with adults [16–21]. Relatively little research on the associations among attention, cognition, development and peripheral BDNF levels have been conducted with children [22–26].

We evaluated the relationships among cognition, attention and peripheral blood BDNF concentrations in children. CNS BDNF crosses the blood-brain-barrier into the peripheral blood [27]. Some reports suggest that serum and cortical BDNF levels are positively correlated and that plasma BDNF levels directly reflect brain tissue levels [28, 29].

In the present study, we have assessed serum and plasma BDNF levels, preschool child IQ, inattention, hyperactivity, internalized/externalized problems, behavioral problems and depression in preschool children (age range 5–7 years).

## Methods

### Participants

Twenty-eight preschool children [13 boys, 15 girls, mean age: 6.16 ± 0.60 years (age range 5–7 years)] who lived in Ulsan metropolitan city in Korea were recruited for this study by advertisements in the Ulsan University Hospital. This study was approved by the Institutional Review Board of the Ulsan University Hospital. In accordance with the Declaration of Helsinki, both the subjects and their parents were advised the procedure. Parents of the subject were required to provide written informed consent prior to participation in the study. The demographic variables of the participants were composed of maternal age at pregnancy, birth weight, paternal education, maternal education, income, and secondhand smoke exposure.

### Psychometric properties

To test intelligence, we administered the Korean Educational Development Institute’s Wechsler Intelligence Scale for Children (KEDI-WISC). The average KEDI-WISC is 100 ± 15 [30].

Sustained attention, vigilance and distractibility were assessed by the computerized continuous performance test (CCPT). The Korean version of the CCPT is a diagnostic tool of attention deficit-hyperactivity disorder and has acquired validity and reliability [31]. CCPT comprises an auditory and visual test that records omission error, commission error, response time and standard deviations of response time. If a T-score is more than 65 in any of these variables, ADHD is suspicious [32].

The children’s color trails test (CCTT) is the children’s version of the color trails test. The CCTT assesses frontal lobe function, including visual-motor coordination, attention, and cognitive flexibility [33]. The Korean version was standardized by Koo and Shin [34]. This test scores the total time to finish (CCTT 1 and CCTT 2) and the difference interference index (total time to finish CCTT 1- total time to finish CCTT 2) [34]. The mean T-score of the CCTT is 50 ± 10 [35]. Higher T-scores indicate better performance on the test [35].

The Stroop color-word test assesses cognitive inhibition and the ability to ignore the interference from irrelevant stimuli [36]. The Korean version of the Stroop color-word test has been standardized [37]. The average T-score of the Stroop color word test is 50 ± 10 [37].

The Korean parent-report version of the child behavior checklist (CBCL) was used to assess child emotional and behavioral problems. This version of the CBCL [38, 39] is a 121-item questionnaire measure which is widely used in Korea. Each item is scored from 0 (absent) to 2 (very often present), and composite scores for each subscale are then converted to give T-scores with a mean of 50 and SD of 10 [38]. Five subscale scores were used to profile results in the present study, namely internalizing problems; externalizing problems; total behavior problems; anxiety/depression and attention problems. The externalizing problems of the K-CBCL comprise attention problems and aggressive and delinquent behavior. The internalizing problems consist of withdrawal; depressed behavior; and somatic complaints [40]. For diagnosis of ADHD, the positive predictive value and specificity of the attention problem section is significant when the child achieves a T-score ≥60, and when the total problem section yields a T-score ≥63 [40].

ADHD screening and symptom severity was assessed by the standardized Korean version of attention deficit hyperactivity disorder rating scale (ARS) [41, 42]. ARS is based on DSM-IV criteria and parent or teacher report. The ARS contains 18 items that include nine inattention relatedness and nine hyperactivity and impulsivity factors. Each item score ranges from 0 (never) to 3 (very often). Therefore, the total range of score is 0 to 54. A reasonable level of sensitivity, specificity and negative predictive value for the diagnosis of ADHD is acquired when the ARS total score is more than 14.5–15.5 [40]. A higher score indicates more severe problems [41].

### Blood BDNF drawing

Blood samples were drawn from all participants at 2 pm. For the serum BDNF analysis, we used a serum separator tube (SST) and allowed samples to clot for 30 min before centrifugation for 15 min at approximately 1000×*g*. The serum was removed, and the separated serum layer was aliquoted into 5-ml polypropylene cryo-vialsand stored at −80 °C until assay analysis. Plasma was collected on ice using EDTA tubes and centrifuged for 15 min at 1000×*g* at 4 °C within 30 min of collection. An additional centrifugation step was conducted on the separated plasma at 10,000×*g* for 10 min at 4 °C, as recommended for complete platelet removal. We removed the plasma, and the separated plasma layer was aliquoted into 5-ml polypropylene cryo-vials and stored at −80 °C until assay analysis. The samples were diluted with diluent included in the R&D Human BDNF Quantikine ELISA kit (Minneapolis, Minnesota) to bring measured levels of BDNF within the range of the standard provided. The results are reported in pg/ml.

### Statistical analyses

All statistical analyses were performed with SPSS version 17.0 for windows. The demographic variables (age, maternal age at onset, birth weight, paternal, maternal education, income, indirect smoking) and psychometric properties (IQ, CCPT, CCTT, Stroop test, CBCL, ARS) of the participants, were ascertained by descriptive statistics. Serum and plasma levels of BDNF were compared to reference values. A two-tailed Pearson χ^2^ test was used to establish the level of correlation between the psychometric scores and serum and plasma BDNF levels. Statistical significance was reported for results above the 0.05 level.

## Results

### Result of variables

Demographic data are shown in Table 1. A total of 28 children [13 boys (46 % of participants) and, 15 girls (54 % of participants)] were recruited. Most parents of the participants had been educated for more than 12 years (82.1 %). The psychometric properties of participants are shown in Table 2. The mean full scale IQ of the participants was 106.89 ± 12.41. The mean CBCL scores were as follows: internalizing problems (45.64 ± 10.31), externalizing problems (48.54 ± 7.34), total behavior problems (46.68 ± 9.55), anxiety/depression (46.64 ± 9.83), and attention problems (43.86 ± 7.71). The mean ARS score was 6.04 ± 5.82. Serum or plasma BDNF levels did not differ statistically across the sexes or with age. Additionally, there were no differences in psychometric scores across the sexes or with age, except for the CCPT commission error (visual) (p = 0.02) and ARS hyperactivity (p = 0.04), inattention (p = 0.005), total (p = 0.03) scores, which showed gender differences.

**Table 1:** Demographic variables of the participants

	Boys (n = 13)	Girls (n = 15)	Total (n = 28)
Age range	5.11–6.80	5.10–7.00	5.10–7.00
Age mean (SD)	6.12 (0.55)	6.19 (0.66)	6.16 (0.60)
Maternal age at pregnancy	29.54 (2.30)	29.73 (3.70)	29.65 (3.07)
Birth weight	3.23 (0.56)	3.04 (0.49)	3.13 (0.52)
Paternal education (n, %)			
12 years	3 (23.1)	3 (20.0)	6 (21.4)
13–16 years	8 (61.5)	12 (80.0)	20 (71.4)
Above 16 years	2 (15.4)	0	2 (7.2)
Maternal education (n, %)			
12 years	2 (15.4)	2 (13.3)	4 (14.3)
13–16 years	8 (61.5)	13 (86.7)	21 (75.0)
Above 16 years	3 (23.1)	0	3 (10.7)
Income (n, %)			
1–3 million/month	3 (23.1)	4 (26.7)	7 (25.0)
3–5 million/month	8 (61.5)	9 (60.0)	17 (60.7)
Above 5 million/month	2 (15.4)	2 (13.3)	4 (14.3)
Indirect smoking			
Yes (n, %)	8 (61.5)	9 (60.0)	17 (60.7)
Serum BDNF level mean (SD) (pg/ml)			
Age 5–6	24,063.33 (10463.72)	21,680.00 (6081.51)	22,871.67 (8253.99)
Age 6–7	19,562.14 (4843.85)	24,938.33 (10309.37)	22,586.25 (8582.34)
Plasma BDNF level mean (SD) (pg/ml)			
Age 5–6	2546.08 (1971.05)	2827.75 (1725.49)	2686.92 (1772.26)
Age 6–7	2728.82 (1605.08)	3205.25 (1682.22)	2996.81 (1612.25)

**Table 2:** Psychometric properties of the participants

	Favorable direction^a^	Minimum	Maximum	Mean (SD)	
				Boys	Girls
IQ					
FSIQ	↑	86	124	106.31 (13.66)	107.40 (11.69)
Verbal	↑	80	127	105.31 (15.55)	105.67 (11.10)
Motor	↑	86	128	105.61 (12.85)	99.87 (28.06)
Computerized continuous performance test					
Visual (T score)					
Omission error	↓	40	135	72.61 (29.91)	60.73 (26.82)
Commission error	↓	40	117	69.46 (21.91)*	52.27 (9.76)*
Auditory (T score)					
Omission error	↓	34	81	55.92 (14.73)	48.93 (11.16)
Commission error	↓	34	61	48.53 (7.03)	46.13 (8.45)
CCTT (T score)					
CCTT 1 time	↑	18	65	35.46 (10.91)	36.67 (14.55)
CCTT 2 time	↑	40	143	85.08 (19.09)	80.93 (23.49)
Inference index	↑	40	67	51.23 (7.36)	52.87 (7.22)
Stroop (T score)					
Inference score	↑	24	61	44.08 (7.79)	47.40 (9.93)
CBCL score (T score)					
Social withdrawal	↓	18	100	47.46 (8.05)	44.80 (30.67)
Somatic complaints	↓	42	65	44.61 (6.66)	47.47 (7.35)
Anxiety/depression	↓	35	68	45.38 (9.07)	47.73 (10.63)
Social problem	↓	37	78	45.00 (8.95)	47.60 (10.38)
Thought problem	↓	45	58	45.92 (3.32)	47.47 (4.29)
Attention problem	↓	35	59	43.62 (7.92)	44.07 (7.79)
Delinquent behavior	↓	40	59	47.08 (6.20)	48.73 (6.42)
Aggressive behavior	↓	37	64	49.31 (6.76)	48.93 (7.96)
Internalizing problems	↓	33	71	44.38 (9.19)	46.73 (11.40)
Externalizing problems	↓	36	64	48.69 (6.75)	48.40 (8.04)
Total behavior problems	↓	30	71	45.38 (8.07)	47.80 (10.82)
ADHD rating scale					
Hyperactivity	↓	0	10	4.92 (3.52)*	2.40 (2.80)*
Inattention	↓	0	13	4.00 (2.16)*	1.40 (2.29)*
ARS total	↓	0	23	8.61 (5.82)*	2.60 (2.56)*

* P < 0.05

^a^Direction in which a change in score indicates good function

### Correlations with peripheral BDNF concentration and psychometric properties

**Fig. 1:**
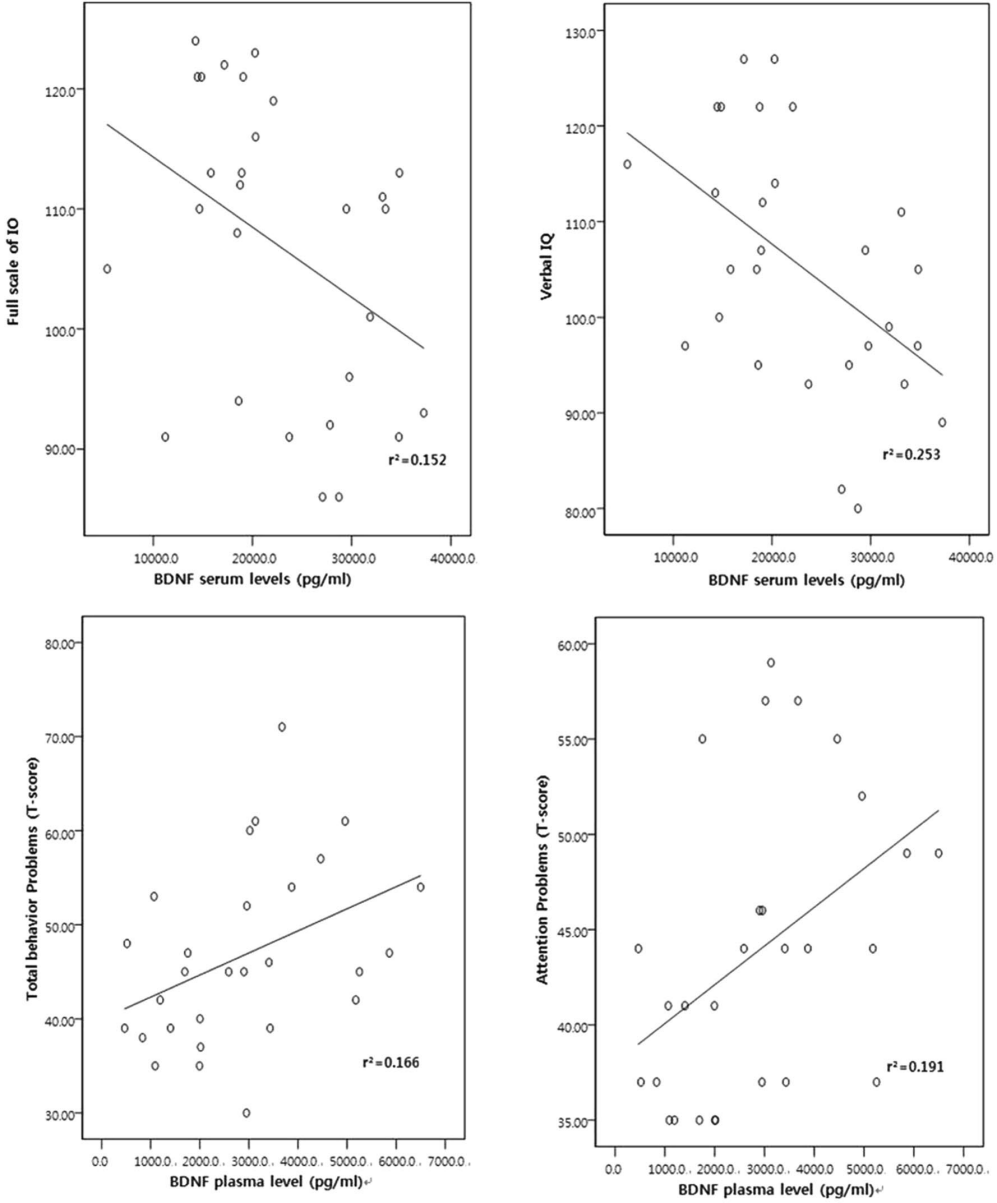
*Scatter plot* of BDNF levels with FSIQ, VIQ, CBCL total problem behavior, and attention problem score

We examined correlations with the Pearson χ^2^ test between the serum and plasma BDNF levels and the full scale IQ (FSIQ), verbal IQ (VIQ), and performance IQ (PIQ) (Table 3). The serum BDNF level was negatively correlated with the FSIQ (r = −0.39, p = 0.04) and VIQ (r = −0.50, p = 0.01), but not with the PIQ (r = −0.12, p = 0.56) (Table 3; Fig. 1). There was no significant relationship between plasma BDNF level and each IQ scale. Additionally, no correlation was found between the serum or plasma level of BDNF and the CCPT, ARS, CCTT or Stroop color-word test (Table 4). The total behavior problem and attention problem sections of the CBCL were positively related to plasma BDNF level [(r = 0.41, p = 0.03), (r = 0.44, p = 0.02)] (Table 5; Fig. 1). No relationship was found between plasma BDNF and social withdrawal, somatic complaints, anxiety/depression, social problems, Thought problems, delinquent behavior, aggressive behavior, internalizing or externalizing problems on the CBCL. No associations were found between serum BDNF level and any CBCL scores.

**Table 3:** Correlation coefficient of IQ with BDNF levels

	Favorable direction^a^	SERUM BDNF	P value	PLASMA BNDF	P value
		Pearson correlation coefficient		Pearson correlation coefficient	
FSIQ	↑	−0.39	0.04	−0.21	0.30
VIQ	↑	−0.50	0.01	−0.02	0.91
PIQ	↑	−0.12	0.56	−0.33	0.08

^a^Direction in which a change in score indicates good function

**Table 4:** Correlation coefficient of ADS, ARS, CCTT, and STROOP with BDNF levels

	Favorable direction^a^	SERUM	P value	PLASMA	P value
		Pearson correlation coefficient		Pearson correlation coefficient	
Computerized continuous performance test (T score)					
Omission error (visual)	↓	0.13	0.51	0.20	0.32
Commission error (visual)	↓	0.03	0.87	−0.19	0.35
Omission error (auditory)	↓	0.01	0.98	0.25	0.19
Commission error (auditory)	↓	0.08	0.70	−0.08	0.67
CCTT (T score)					
CCTT 1 time	↑	0.18	0.36	−0.05	0.79
CCTT 2 time	↑	0.19	0.35	0.01	0.96
Inference index	↑	−0.13	0.53	−0.10	0.60
Stroop test (T score)					
Inference score	↑	0.02	0.90	0.19	0.34
ARS	↑				
Hyperactivity	↓	−0.13	0.50	0.20	0.30
Inattention	↓	−0.10	0.62	0.29	0.13
ARS total	↓	−0.11	0.59	0.28	0.16

^a^Direction in which a change in score indicates good function

**Table 5:** Correlation coefficient of CBCL with BNDF levels

	Favorable direction^a^	SERUM	P value	PLASMA	P value
		Pearson correlation coefficient		Pearson correlation coefficient	
Social withdrawal (T score)	↓	−0.17	0.34	0.18	0.35
Somatic complaints (T score)	↓	0.05	0.80	0.27	0.17
Anxiety/depression (T score)	↓	−0.13	0.51	0.36	0.06
Social problem (T score)	↓	−0.08	0.97	−0.13	0.51
Thought problem (T score)	↓	−0.05	0.81	0.02	0.93
Attention problem (T score)	↓	−0.02	0.94	0.44	0.02
Delinquent behavior (T score)	↓	0.06	0.77	0.12	0.53
Aggressive behavior (T score)	↓	−0.01	0.95	0.37	0.05
Internalizing problems (T score)	↓	−0.06	0.75	0.37	0.06
Externalizing problems (T score)	↓	−0.04	0.84	0.29	0.13
Total behavior problems (T score)	↓	−0.05	0.81	0.41	0.03

^a^Direction in which a change in score indicates good function

## Discussion

This study is the first to investigate the association between BDNF levels and preschool children’s cognitive development in healthy subjects. We found that serum BDNF level was negatively associated with both full-scale and verbal IQ scores and that plasma BDNF level was negatively associated with CBCL attention and behavior problem scores.

BDNF is an important factor in neuro-development [3, 4]. Our results show that BDNF may play a role in intelligence, attention and clinical symptoms of preschool children with neuro-developmental disorders such as intellectual disability and ADHD. Higher peripheral BDNF concentration could be a biomarker of these states.

There are some reports of an association between BDNF and intellectual disability and of a general inverse correlation with intelligence in children [23–25]. Nelson et al. [23] reported elevated peripheral blood BDNF levels in neonates with intellectual disabilities than in controls. They suggested that BDNF dysregulation may play a role in the development of intellectual disability and that BDNF levels may be an early biomarker for identification of intellectual disability [23]. Miyazaki et al. [24] also found that children and adolescents (mean age: 11.0 ± 5.9 years), diagnosed with an intellectual disability, had higher blood BDNF levels than controls. They concluded that elevated BDNF levels may reflect an abnormal state in prenatal or early postnatal neuronal development [24]. However, Taurines et al. [25] found no correlation between altered peripheral BDNF mRNA expression and BDNF protein concentrations in blood of children and adolescents with autism spectrum disorder.

Research has been conducted on cognitive function of BDNF over-expressed transgenic mice [10, 43]. Croll et al. [43] found that BDNF over-expressed transgenic mice show significant impairment in learning (passive avoidance) and increase locomotor activities (maze arm entries) and hyper-excitability in the CA3 area of the hippocampus. They suggested that excess BDNF may interfere with normal learning and memory, and this result is due to too much excitability in the learning circuit or too much plasticity leading to synaptic refinement [43]. Cunha et al. [10] also described that overexpression of BDNF in the forebrain may reduce learning and memory formation in mice. They proposed that the physiological amount of BDNF is helpful in learning and memory, but an increased or decreased level of BDNF induces inhibitory and excitatory neurotransmission in the brain, causing loss of synaptic refinement and impairment of learning and memory [5].

Some researchers found a relationship between a polymorphism of the BDNF gene and cognitive functions in humans [44–46]. Egan et al. [44] reported that the Val66Met polymorphism of the BDNF gene, valine (Val) to methionine (Met) substitution at codon 66, is related to poor episodic memory, abnormal hippocampal activation, abnormal intracellular trafficking and dysregulation of BDNF secretion in humans. fMRI research of the Val66Met polymorphism of the BDNF gene also described that the Val66Met polymorphism impacts memory related brain activity in the healthy humans. Additionally, the Met allele of the BDNF Val66Met polymorphism is related to increased serum BDNF levels in adults [46]. Therefore, we need additional research about single nucleotide polymorphisms of the BDNF gene in children with higher serum levels of BDNF such as those in this study.

There are some controversial results about the relationship between BDNF and ADHD [20, 22, 47]. Shim et al. [22] found that children (mean age: 8.8 ± 2.3 years), who are diagnosed with ADHD, have higher plasma BDNF levels than control children, and the severity of inattention problems have a positive correlation with plasma BDNF levels. They suggested that increased BDNF levels possibly reflect a compensatory mechanism in the response of abnormal and late brain maturation [22]. However, Scassellati et al. [47] found no difference in serum BDNF level between ADHD children (mean age: 8.8 ± 2.3 years) and control children. Corominas-Roso et al. [20] reported that adults with ADHD (mean age: 33.43 ± 8.99 years) have lower BDNF levels than control adults. They suggested that low BDNF levels may contribute to the neurodevelopmental deficit in ADHD [20]. A study have reported that the serum BDNF level increases over the first several years and, then decreases after reaching adult levels in humans [26]. Therefore, more research is needed on the association between peripheral BDNF concentration and neuro developmental disorders in human development.

Animal studies have also reported controversial results about the relationship of BDNF with inattention and hyperactivity [43, 48–50]. Young adult transgenic mice, which over-express BDNF, have a tendency to spend more time being mobile [43], but BDNF knockout adult mice demonstrate more impulsive behavior, hyperactivity and learning deficiency [48–50].

Some studies have reported on the association between the BDNF gene and ADHD [51–53]. Of these studies, a cohort study on the association between the Val66Met polymorphism of BDNF and children with ADHD found that the Met allele is associated with ADHD symptoms, such as hyperactivity-impulsivity [53]. Another study found that the Valine allele of the Val66Met polymorphism of the BDNF gene is associated with the pathogenesis of ADHD [52]. Thus, additional studies are needed on the association between peripheral BDNF concentration and single nucleotide polymorphisms of the BDNF gene in children with ADHD.

Our study used serum and plasma levels of BDNF to investigate the relationships among peripheral blood BDNF level and childhood IQ and neurobehavior. In this study, serum BDNF level was related to VIQ and FIQ. However, plasma BDNF was not associated with VIQ and FIQ. Plasma BDNF levels were related to externalizing problems and attention problems according to the CBCL, but not with serum BDNF levels. Many other studies have assessed the relationship between the serum or plasma level of BDNF and neuropsychiatric or developmental disorders [16, 19, 20, 22, 24]. However, there is still no standard method to measure peripheral BDNF levels. Additionally, the relationship between serum and plasma BDNF levels has not established. Yoshimura et al. [54] reported that plasma and serum levels of BDNF are positively correlated in healthy volunteers. However, Bocchio-Chiavetto et al. [55] found no correlation between plasma and serum levels of BDNF in major depressive patients in a meta-analysis. Some researchers have suggested that plasma BDNF is a reliable indicator of brain BDNF levels because of the little influence of the BDNF that is stored in platelets [22, 56]. Other researchers have suggested that serum BDNF is a valid marker of brain BDNF because serum BDNF reflects the BDNF accumulated by platelets during illness or treatment periods [57]. Accordingly, we used two indicators, serum and plasma BDNF. Therefore, to use BDNF as a biomarker, a standardized method of measurement of BDNF and the source of peripheral BDNF is needed.

This study has some limitations that must be considered. First, we did not assess our subjects with structured interviews to rule out psychiatric illnesses. However, we assessed their intelligence and psychiatric history using a standardized intelligence scale and questionnaire. Second, we assessed the correlation between peripheral blood BDNF and intelligence and psychiatric problems in the same group. Therefore, we could not compare the absolute peripheral BDNF level of patients with ADHD or other DSM-5 neurodevelopmental disorders. Third, this study was a cross-sectional study. In BDNF over-expressing mice, memory retention was impaired in younger animals, but not in older ones [10]. Thus, a long-term follow up study on blood BDNF levels and psychopathologies is needed. Last, we included 28 preschool children, and higher number of subjects would increase statistical power.

## Conclusions

Our results suggest that high peripheral concentration of BDNF is related to intelligence, inattention and behavioral problems. Further studies on BDNF metabolism are required, using a standardized measurement method for BDNF ascertainment, with parallel genetic analysis of BDNF gene polymorphisms, with a robust sample size and with long-term follow-up are needed to further validate this line of research and to clarify the role and relevance of differences in peripheral BDNF as a potential biomarker.

## Abbreviations

ADHD: attention-deficit hyperactivity disorder
ARS: ADHD rating scale
BDNF: brain-derived neurotrophic factor
CBCL: child behavior check list
CCPT: computerized continuous performance test
CCTT: children’s color trail test
FSIQ: full scale IQ
PIQ: performance IQ
STROOP: Stroop color-word test
VIQ: verbal IQ
WISC: Wechsler Intelligence Scale for Children
